# Resolving Block Screw Obstruction in Tibial Intramedullary Nailing

**DOI:** 10.7759/cureus.77997

**Published:** 2025-01-26

**Authors:** Nizar Ismail, Alwyn Abraham

**Affiliations:** 1 Trauma and Orthopaedics, East Midlands Deanery, Leicester, GBR; 2 Trauma and Orthopaedic Surgery, University Hospitals of Leicester National Health Service (NHS) Trust, Leicester, GBR

**Keywords:** blocking screw, intramedullary nailing of the tibia, poller screw, tibia nailing, tibia shaft fracture

## Abstract

This technical report describes a new solution for dealing with intramedullary nail blockage during tibial fracture operation. It discusses a case in which a screw obstructed nail insertion, a complication encountered during the procedure, and outlines the development of a four-step minimally invasive technique.

The obstruction was localized using fluoroscopy, and a unicortical drill hole (4.5 mm) was created directly above the blocking screw. A ring handle spike instrument was introduced through the hole to act as a lever, allowing the nail to advance beyond the obstruction. Final fluoroscopic confirmation was then performed to verify the proper position of the nail.

This approach successfully achieved adequate nail placement without compromising the reduction of the fracture or significantly increasing the operative time, all while preserving the minimally invasive nature of the procedure.

## Introduction

Tibia bone fractures represent about 17% of all lower extremity fractures and 4% of all fractures in Medicare patients. These injuries show a bimodal age distribution, typically affecting young patients through high-energy trauma and older individuals through lower-energy mechanisms like falls, with male patients being more frequently affected than female patients. When considering anatomic location, fractures of the proximal third of the tibia comprise 5-10% of all tibial shaft fractures [1]. 

Intramedullary nailing is a commonly used and successful treatment for tibial shaft fractures [2]. This procedure has numerous benefits, including faster recovery times, enhanced stability, and minimally intrusive approaches. Blocking screws, also named poller screws, are often used in intramedullary nailing to guide the nail to aid in fracture reduction [3,4]. 

Poller screws need to be correctly placed to create a corridor for the intramedullary nail. It effectively narrows the medullary cavity, opposing shearing forces and enhancing compression forces, favoring the fracture's union. According to a systematic review, the complications associated with intramedullary nails in the tibia, when combined with block screws, were low. The rates of complications included nonunion (4%), coronal plane malunion (6%), deep infections (5%), superficial infections (6%), and the need for secondary procedures (8%) [5,6]. 

In this study, we describe a case where a blocking screw ultimately prevented the insertion of a tibial nail by obstructing its passage. To address this challenge, we created a novel maneuver to drill a unicortical hole and push the nail around the obstruction. In the following paragraphs, we share the problem we encountered, the reasoning that led us to the solution, and the technical approach we found to solve this issue.

## Technical report

Initial procedure

The intramedullary nail procedure was done following the Arbeitsgemeinschaft für Osteosynthesefragen (AO) protocol [7]. The patient was planned to have a standard intramedullary nailing of a tibial shaft fracture. The entry point was established after positioning the patient, preparing and draping the patient, and using a sterile triangle. The tibia bone was reamed, and the nail was inserted. Blocking screws were used to aid fracture reduction.

Encountered complication

Upon trying to insert the intramedullary nail, we encountered significant resistance. Fluoroscopy showed that the previously inserted poller screw had blocked the nail pathway. Many standard techniques were tried, including manipulating the fracture in different directions, but the nail could advance no further. At this point, we acknowledged the need for a different solution, so we devised the following to resolve the situation:

Step 1

**Figure 1:**
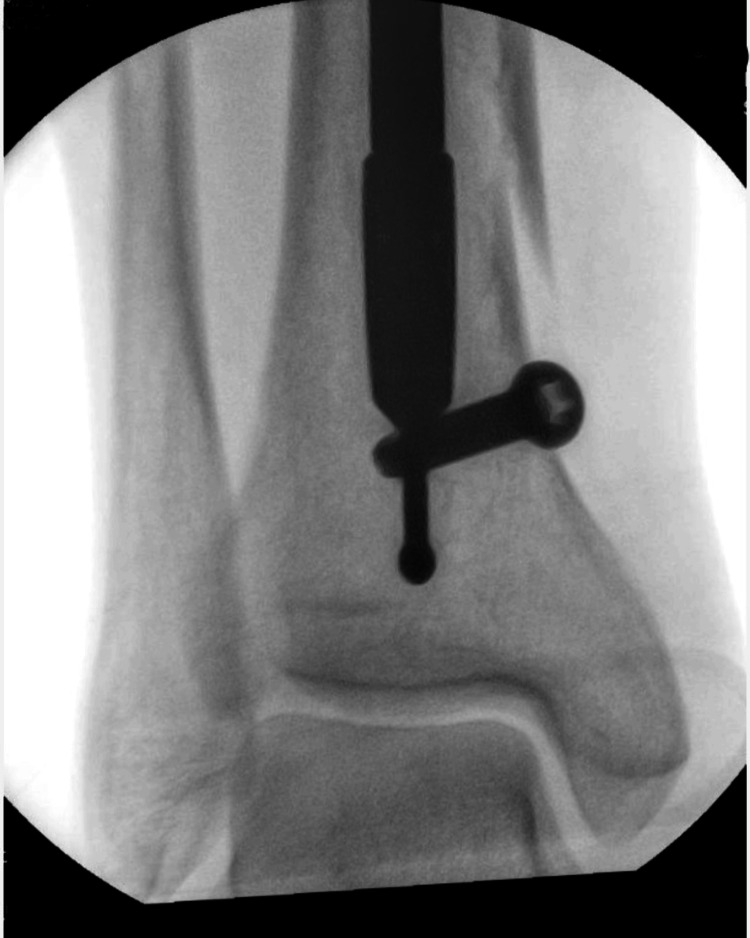
An X-ray showing the nail obstructed by the poller screw.

Fluoroscopic guiding allowed for a precise location of the obstruction (Figure 1).

Step 2

A 4.5 mm drill bit was used to drill a unicortical hole in the tibia. This hole was purposely located over the lowest part of the nail's trajectory and above the blocking screw.

Step 3

**Figure 2:**
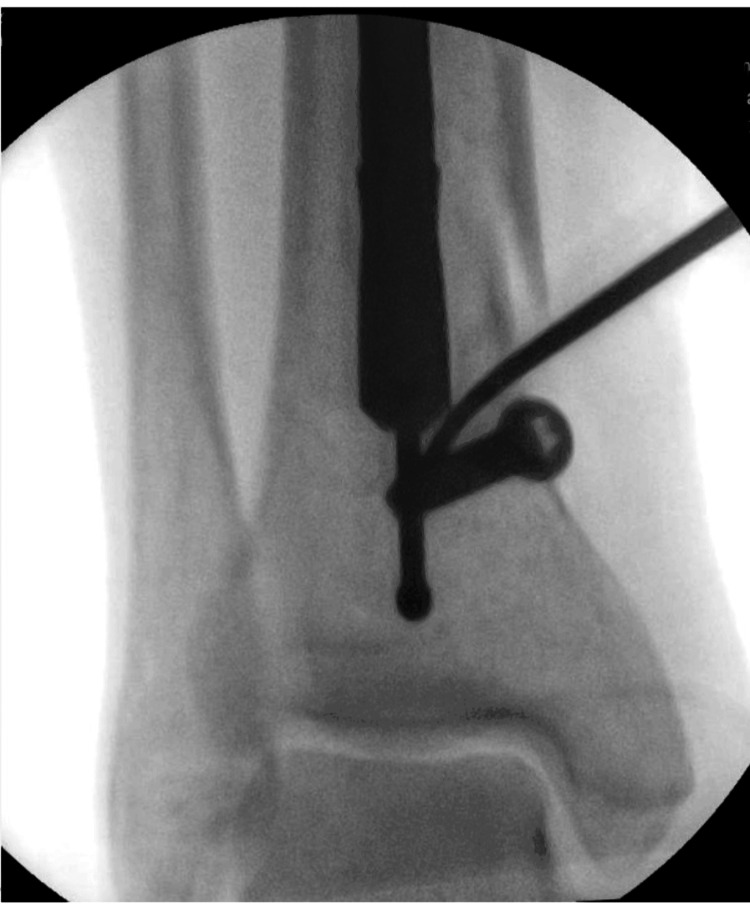
The ring handle spike instrument was inserted through the hole drilled by a 4.5 mm drill to manipulate the nail, allowing it to bypass the screw.

**Figure 3:**
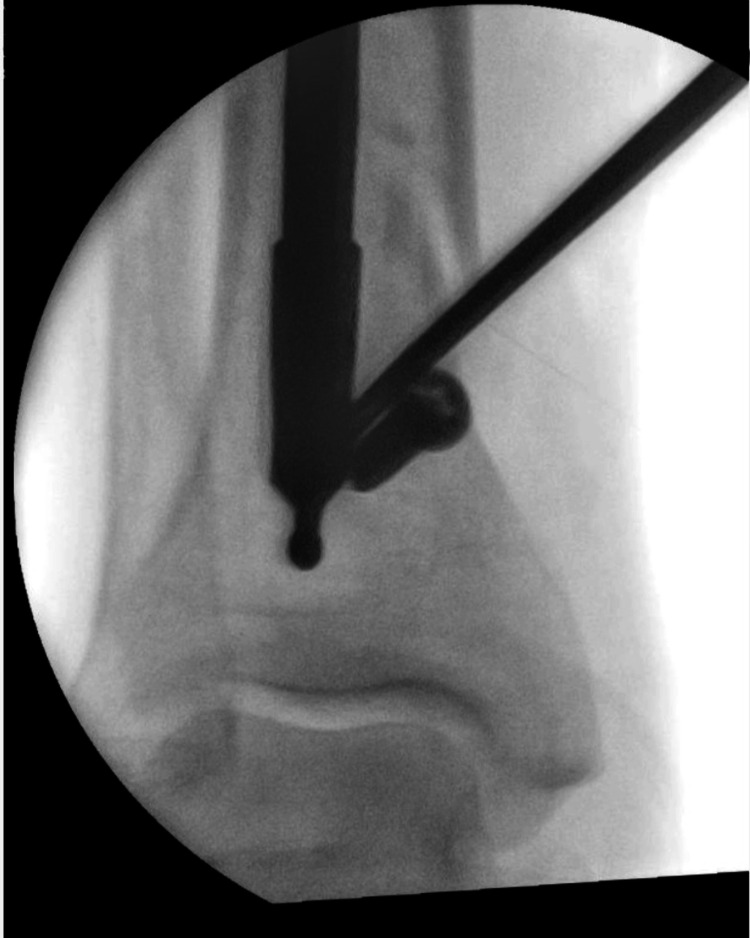
An X-ray demonstrating the nail being leveraged over the screw.

A ring handle spike instrument was inserted into the new hole and used as a lever to move the nail. Using the ring handle spike to apply controllable force, we allowed the nail to slide over the poller screw (Figures 2-3).

Step 4

**Figure 4:**
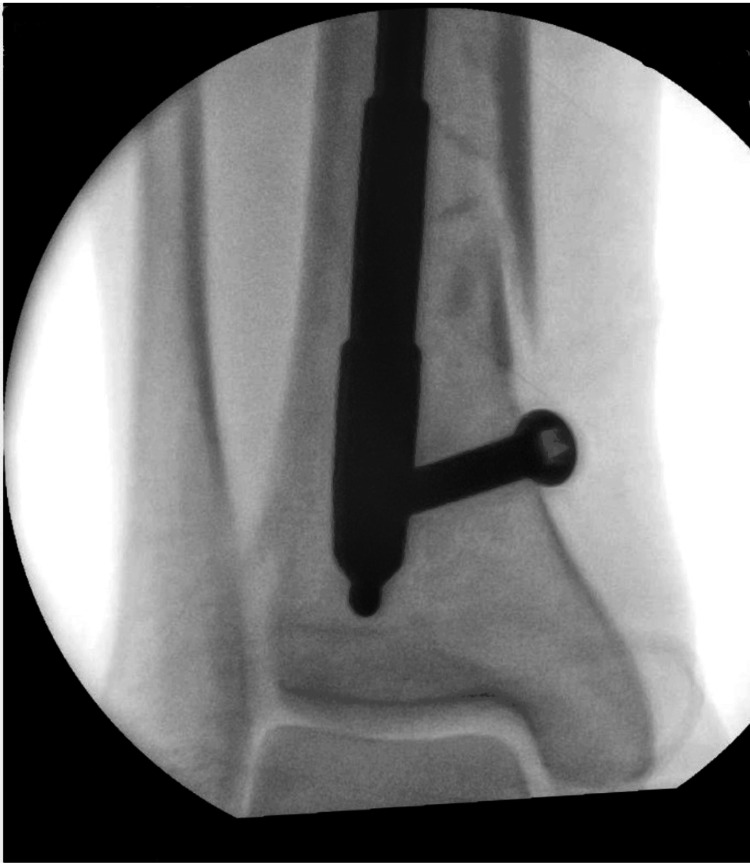
An X-ray illustrating the nail's final position after manipulation.

Confirmation: Fluoroscopy confirmed the correct nail position (Figure 4).

**Figure 5:**
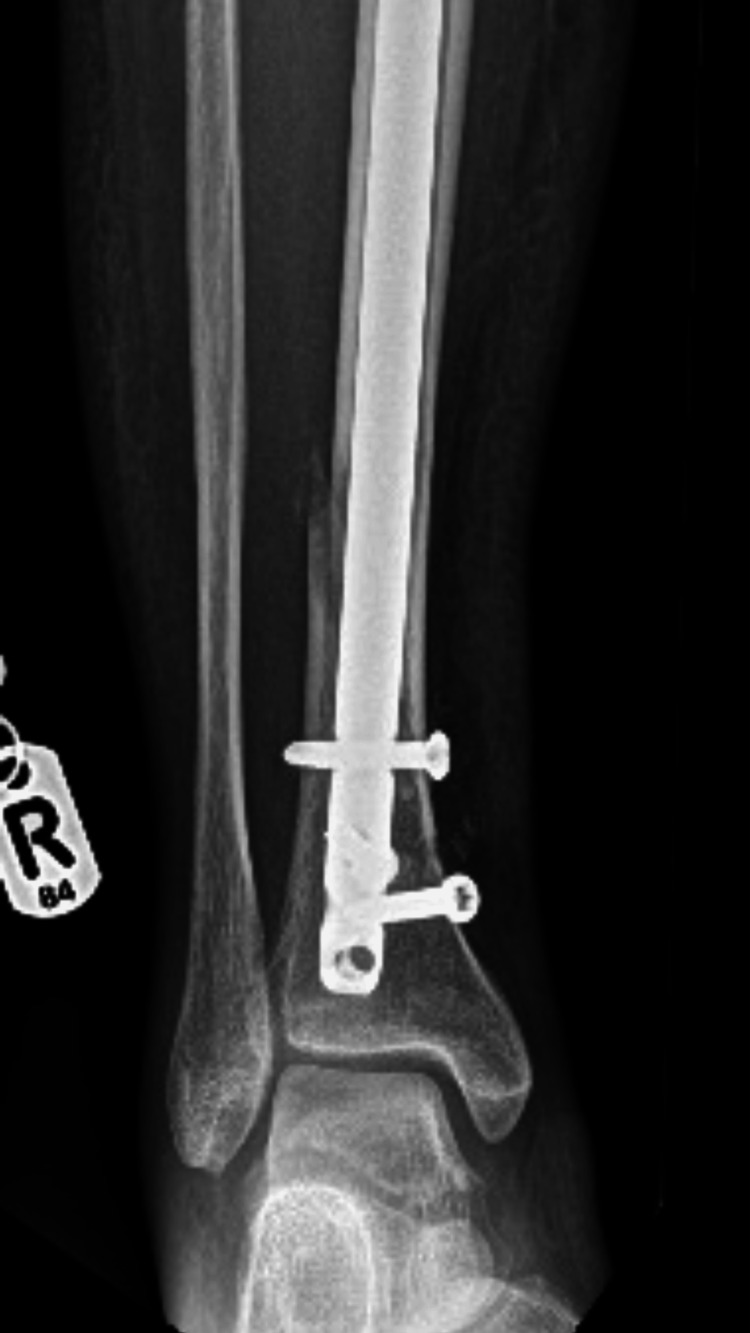
A postoperative X-ray from the anterior-posterior (AP) view.

**Figure 6:**
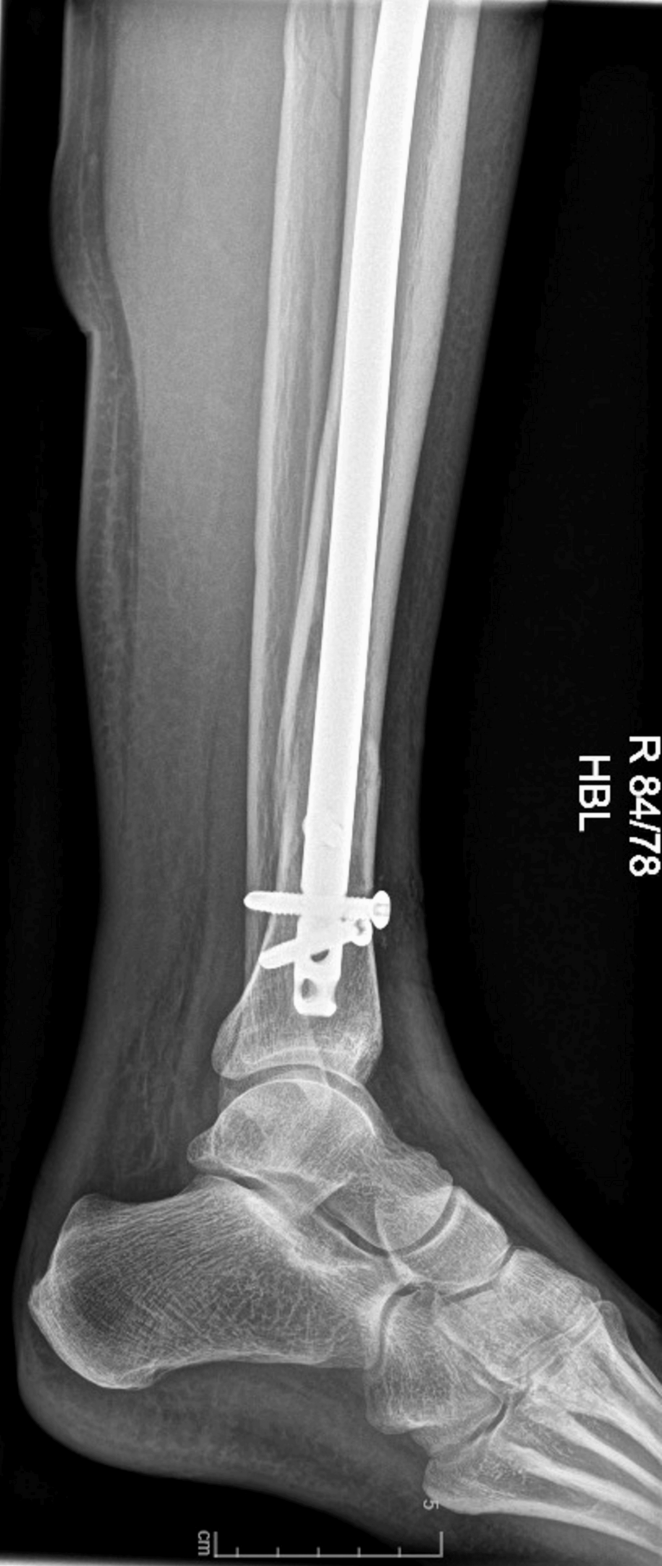
A postoperative X-ray from the lateral view.

Using this technique, we were able to navigate the intramedullary nail past the blocking screw. We managed to deliver the nail to its final position smoothly. Imaging performed after the procedure confirmed the correct nail position and fracture reduction (Figures 5-6).

## Discussion

Block screws help fractures and minimize displacement, but occasionally, they can prevent the nail from passing the medullary canal. The issue may result in longer surgical times, more radiation exposure, and even fractures during the surgery if not appropriately addressed [2,6].

The primary advantage is its simplicity and minimal additional tissue trauma. We maintained the minimally invasive nature of intramedullary nailing by utilizing existing instruments and creating only a unicortical drill hole. This approach aligns with findings from Hipp et al. who demonstrated that small cortical holes do not significantly compromise bone biomechanics regarding strength [8].

The leveraging maneuver is a well-known method in orthopedic surgery for reducing fractures. It is used for different types of fractures. The Kapandji technique is an example and has been used to reduce distal radius fractures since 1976 [9]. 

The role of blocking screws in tibial nailing remains well-established for controlling alignment, particularly in metaphyseal fractures. Research by Tennyson et al. demonstrated a significant improvement in alignment when using blocking screws and a reduction in risks of malunion and nonunion; our technique preserves these benefits while offering a solution when block screws become problematic [4,6].

Hannah et al. suggested a novel approach for optimal blocking screw placement techniques and recommended careful preoperative planning with consideration of nail diameter and trajectory. When prevention fails and the poller screw obstructs the nail, our technique provides a viable alternative to more invasive solutions, such as screw removal or additional surgical approaches [10].

The successful management of blocked intramedullary nail insertion represents a significant technical challenge during surgery. Our described technique of unicortical drilling and leveraging offers a practical solution when encountering blocking screw obstruction. This technical report constitutes 'Level 4' evidence, yet it provides a base for more advanced research. Larger studies will be helpful to better validate this procedure of the technique and its results. 

## Conclusions

The technique presented in this work provides a practical solution to the difficult circumstance in which a blocking screw prevents tibial nail entry during intramedullary nailing. Using an adequately placed unicortical drill hole and leveraging the nail with a ring handle spike, surgeons can solve this challenge without sacrificing fracture reduction or considerably increasing operational time.
